# Comparative evaluation of a surface‐based respiratory monitoring system against a pressure sensor for 4DCT image reconstruction in phantoms

**DOI:** 10.1002/acm2.14174

**Published:** 2023-10-10

**Authors:** Abdallah Qubala, Jehad Shafee, Vania Batista, Jakob Liermann, Marcus Winter, Daniel Piro, Oliver Jäkel

**Affiliations:** ^1^ Heidelberg Ion Beam Therapy Center (HIT) Heidelberg Germany; ^2^ Faculty of Medicine University of Heidelberg Heidelberg Germany; ^3^ National Center for Radiation Research in Oncology (NCRO) Heidelberg Institute of Radiation Oncology (HIRO) Heidelberg Germany; ^4^ Saarland University of Applied Sciences Saarbruecken Germany; ^5^ Department of Radiation Oncology Heidelberg University Hospital Heidelberg Germany; ^6^ National Center for Tumor Diseases (NCT) Heidelberg Germany; ^7^ Department of Medical Physics in Radiation Oncology German Cancer Research Center (DKFZ) Heidelberg Germany

**Keywords:** 4DCT reconstruction, breathing detection, breathing surrogate, commissioning, mobile tumors, motion artifacts, respiratory monitoring system, Surface‐guided radiotherapy

## Abstract

Four‐dimensional computed tomography (4DCT), which relies on breathing‐induced motion, requires realistic surrogate information of breathing variations to reconstruct the tumor trajectory and motion variability of normal tissues accurately. Therefore, the SimRT surface‐guided respiratory monitoring system has been installed on a Siemens CT scanner. This work evaluated the temporal and spatial accuracy of SimRT versus our commonly used pressure sensor, AZ‐733 V.

A dynamic thorax phantom was used to reproduce regular and irregular breathing patterns acquired by SimRT and Anzai. Various parameters of the recorded breathing patterns, including mean absolute deviations (MAD), Pearson correlations (PC), and tagging precision, were investigated and compared to ground‐truth. Furthermore, 4DCT reconstructions were analyzed to assess the volume discrepancy, shape deformation and tumor trajectory.

Compared to the ground‐truth, SimRT more precisely reproduced the breathing patterns with a MAD range of 0.37 ± 0.27 and 0.92 ± 1.02 mm versus Anzai with 1.75 ± 1.54 and 5.85 ± 3.61 mm for regular and irregular breathing patterns, respectively. Additionally, SimRT provided a more robust PC of 0.994 ± 0.009 and 0.936 ± 0.062 for all investigated breathing patterns. Further, the peak and valley recognition were found to be more accurate and stable using SimRT. The comparison of tumor trajectories revealed discrepancies up to 7.2 and 2.3 mm for Anzai and SimRT, respectively. Moreover, volume discrepancies up to 1.71 ± 1.62% and 1.24 ± 2.02% were found for both Anzai and SimRT, respectively.

SimRT was validated across various breathing patterns and showed a more precise and stable breathing tracking, (i) independent of the amplitude and period, (ii) and without placing any physical devices on the patient's body. These findings resulted in a more accurate temporal and spatial accuracy, thus leading to a more realistic 4DCT reconstruction and breathing‐adapted treatment planning.

## 1 INTRODUCTION

Breathing‐induced motion of the target volume and organs at risk (OARs) is an important source of intra‐fractional patient geometry variations,^1^, ^2^, ^3^ especially during three‐dimensional computed tomography imaging (3DCT) of thorax and abdomen in free breathing.^4^, ^5^ Compared to static tumor targets, such variations may complicate treatment planning and dose delivery in radiotherapy (RT) regarding motion and image artifacts.^6^, ^7^, ^8^ These artifacts can result in distortions when assessing the shape, trajectory, volume and Hounsfield unit of the tumor target and OARs.^4^, ^7^, ^9^ As a result, an undesired dose distribution may be applied to the target volume and OARs, especially when implementing tumor treatment approaches that use high doses with extremely steep dose gradients.^10^, ^11^, ^12^

Various treatment strategies are clinically implemented to account for intra‐fractional tumor motion during treatment planning and dose delivery, such as (i) increasing the internal margin of the target volume using the internal target volume (ITV) concept,^13^, ^14^ (ii) using abdominal compression to restrict the tumor motion (e.g., liver treatments),^2^ (iii) breath‐hold methods,^2^, ^15^ (iv) tumor tracking,^15^, ^16^ and (v) utilizing breathing‐adapted gating approaches to attempt tracking of patient breathing, and irradiating the tumor during specific breathing phases.^2^, ^17^, ^18^, ^19^ For the latter,^4^, ^6^, ^20^, ^21^, ^22^ four‐dimensional computed tomography (4DCT) is used, which allows image reconstruction at specific breathing phases based on either the amplitude or the phase angle.^4^, ^5^, ^23^, ^24^, ^25^ Different respiratory monitoring systems (RMSs), such as pressure sensors, skin surface cameras, radiofrequency‐based systems, and fiducial markers with image‐guided RT, are used to measure breathing patterns.^2^, ^26^, ^27^, ^28^, ^29^, ^30^

In our department of radiation oncology at the Heidelberg university hospital (Heidelberg, Germany), the respiratory gating system AZ‐733 V (Anzai Medical Co., Ltd., Shinagawa, Tokyo),^7^, ^22^, ^31^ is used for 4DCTs. The disadvantages of using Anzai include that the recording location of the breathing pattern on the skin surface is only based on one point, it is sometimes non‐reproducible, and depends on the strength of the belt fixation (too loose, good or too tight), which may distort the 4DCT reconstruction. In addition, the recorded breathing pattern is a non‐quantitative breathing signal based on a relative variation resulting from the minimum and maximum pressure values. Accurate spatial and temporal breathing signals acquired from the RMSs may significantly reduce the motion artifacts in the 4DCT and avoid inaccurate reconstructions of the OARs and ITV, leading to accurate tumor trajectory assessment and dose delivery.^6^, ^22^ For this purpose, an optical surface‐guided radiotherapy (SGRT) system, the SimRT, (developed by VisionRT Ltd, London, United Kingdom) has been recently installed at one of our two CTs in our department of radiation oncology which shows the spatial deviation of the patient breathing on the skin surface as an external surrogate.

In the last decade, SGRT has been investigated intensively^27^, ^32^, ^33^, ^34^ to ensure correct patient positioning and tracking during an RT course without additional doses. Furthermore, SGRT may supply a faster and more precise patient positioning compared with skin marks, reducing the re‐imaging rate in the clinical patient setup workflow. For this aim, it compares the current and reference patient skin surface within a user‐defined region of interest or a predefined patch.

In this work, we present a comparative performance assessment of SimRT versus Anzai to evaluate the reproducibility, spatial, and temporal accuracy of the breathing patterns and quality of reconstructed mobile tumors in 4DCT images. The measurements were performed using a dynamic thorax phantom and following international guidelines.^2^, ^28^, ^29^, ^35^ Various parameters of the recorded breathing patterns, including the breathing amplitude, mean absolute deviation (MAD), Pearson correlation (PC), tagging precision, sampling rate, and period, were investigated and compared to ground‐truth. Additionally, 4DCT reconstructions with different breathing amplitude values were evaluated for their tumor volume discrepancy, tumor shape deformation, and tumor trajectory error. Finally, the baseline drift of the breathing patterns on the amplitude scale during image acquisition was also investigated.

## 2 METHODS AND MATERIALS

### 2.1 Siemens CT scanner

**FIGURE 1:**
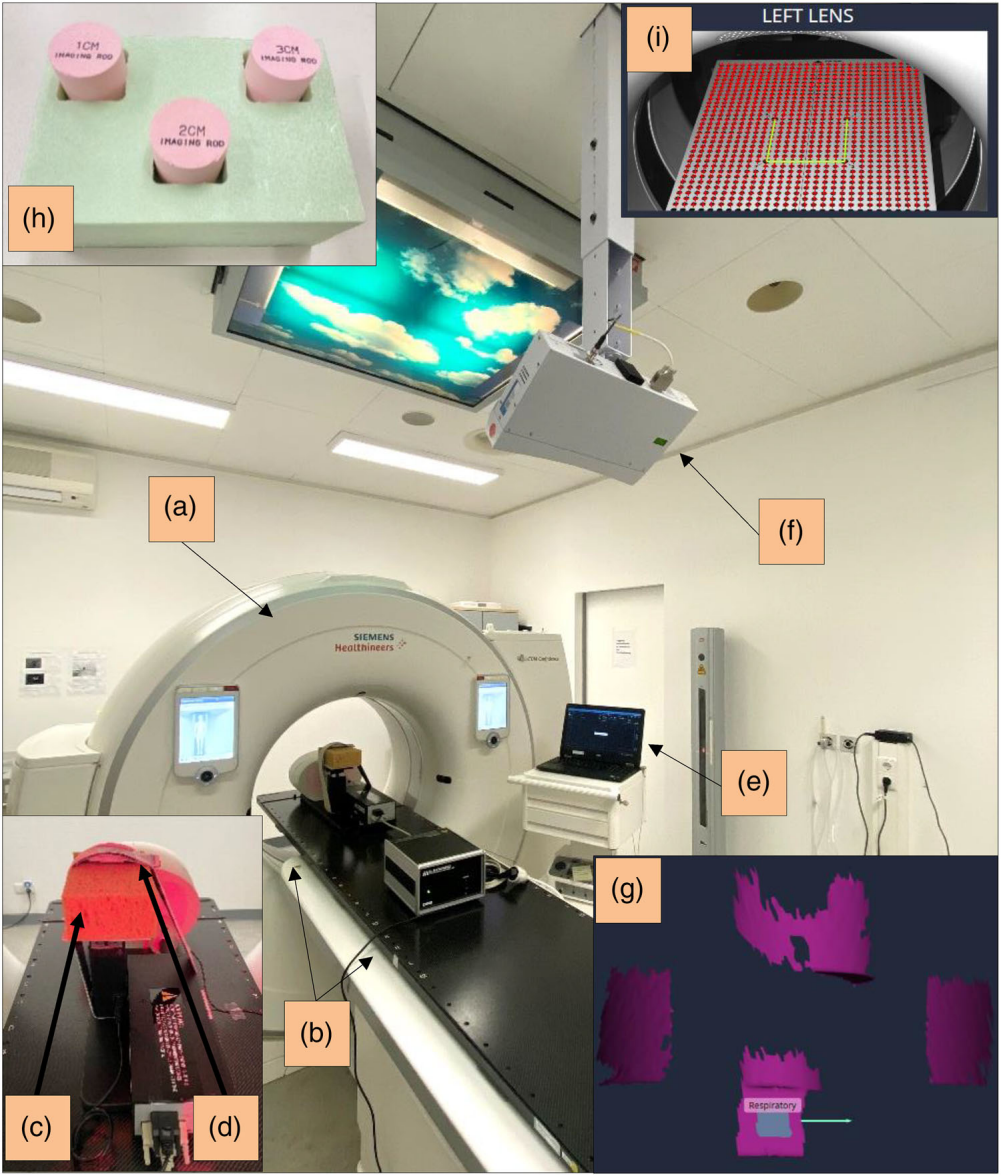
Experimental setup at the Somatom Confidence CT room (a) including the CIRS Phantom (b), sponge (c), Anzai belt (d), Anzai laptop (e), SimRT camera pod (f), reference capture with patch in grey (g), styrodur block in green including three imaging rods in pink (h), and the calibration plate (80 × 60) cm (i). CT, computed tomography; CIRS, Computerized Imaging Reference Systems.

All measurements were performed on the SOMATOM Confidence helical multi‐slice CT scanner (Siemens Healthineers, Erlangen, Germany) which is used for the treatment planning CT simulation (Figure 1). The CT image sorting technique used for 4DCT studies is the phase‐based sorting algorithm^23^ which supplies the image reconstruction at specific breathing phases according to the breathing patterns recorded by the RMS to reduce breathing‐induced motion artifacts and provide an explicit trajectory of the tumor in free breathing.^1^ An example of 3DCT/4DCT parameters used in our institution for the abdominal region is presented in Table 1.

**TABLE 1:** The CT parameters on the Somatom Confidence CT scanner used for this work.

CT Parameter	Static 3DCT	Dynamic 4DCT
**Voltage (kV)**	120	120
**Effective current (mAs)**	300	50
**Slice collimator (mm)**	1.2 × 16	1.2 × 16
**Slice thickness (mm)**	3	3
**Rotation time (s)**	1	0.5
**Pitch**	0.85	0.09
**Reconstruction kernel**	Br40	Br38
**FOV (mm)**	500	500

Abbreviations: 3D, 3‐dimensional; 4D, 4‐dimensional; CT, computed tomography; kV, kilo voltage; mAs, milli ampere second; mm, millimetre; s, second.

### 2.2 RMSs

The Anzai system is utilized with a 40 Hz fixed sampling rate and serves as a surrogate system for the breathing amplitude. It consists of a pressure sensor (diameter of 30 mm and thickness of 9.5 mm) attached to a belt pocket, which can be fixed at a body site (abdomen or breast). By calculating the pressure caused by the body volume variation, the breathing pattern can be obtained and transferred in real time into the CT console. The breathing phases are tagged through a predictive algorithm in the software.

The second system, SimRT, consists of a single pod with two image sensors and a projector that displays optical random speckle patterns on the patient's body surface with a field of view (FOV) of 20 cm × 50 cm in the isocentre plane. Based on a fixed 5 cm × 5 cm patch, SimRT calculates the real‐time position deviations of a surface region by using a rigid‐body transformation between the reference and the current surface. The principles used by the software are active stereo photogrammetry and triangulation.^32^, ^36^ Moreover, the pod is mounted on the ceiling above the foot of the couch with a view into the CT‐bore and can be used for several applications such as 4DCT or deep inspiration breath hold.^37^ SimRT has an automatic learning period before getting ready to detect the breathing pattern which can be reviewed and imported into the CT console retrospectively. Both Anzai and SimRT can be connected with an interface to the CT scanner and receive the beam on/off status.

### 2.3 Breathing phantom

**FIGURE 2:**
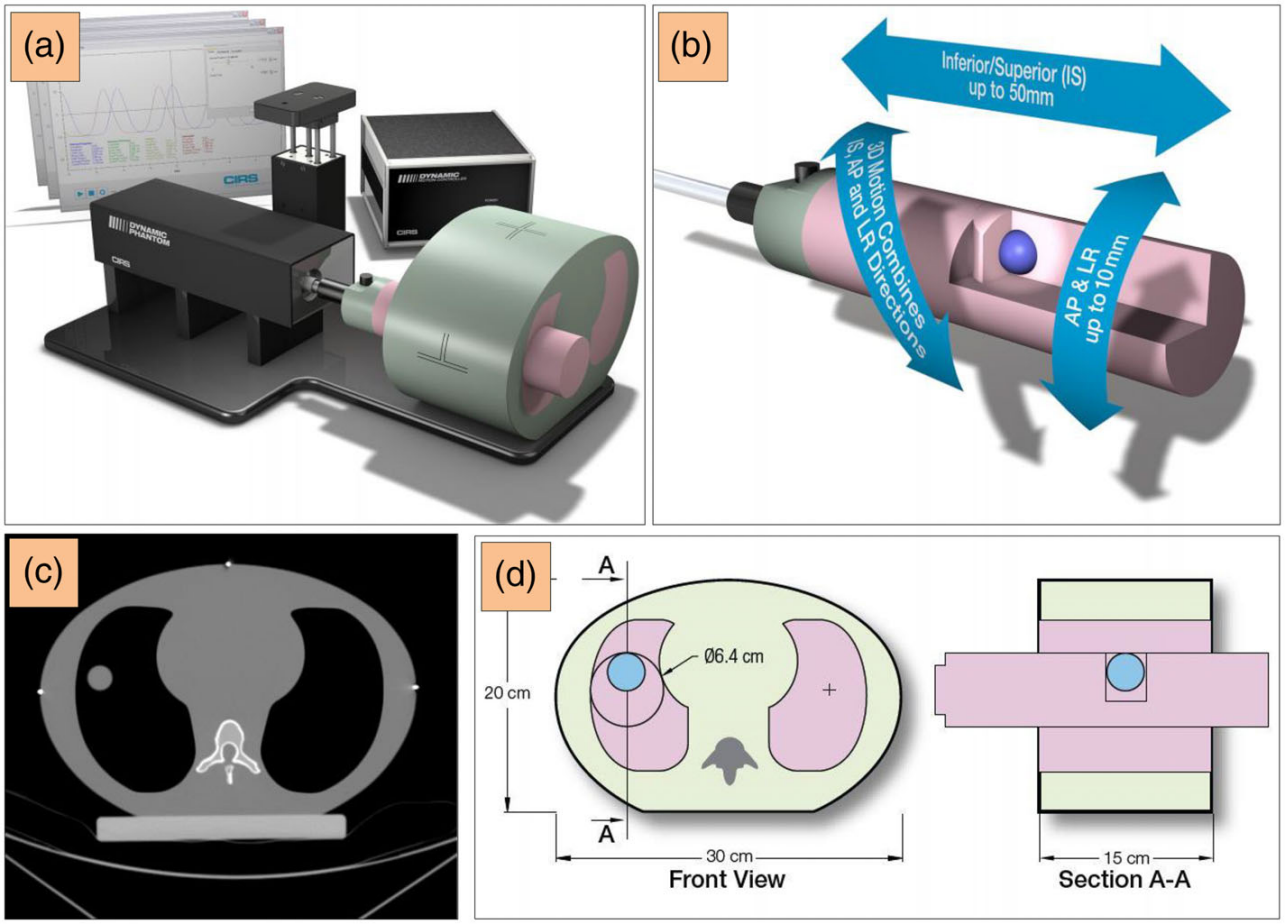
The CIRS dynamic thorax phantom (a) has three possible motion directions of the rod which has an insert with a tumor target (b). An axial CT image to show the internal tissue‐equivalent modules (c), and a cross‐section of the internal structures using the imaging insert (d) used in this work. A motion uncertainty of ± 0.1 mm can be achieved.^38^ AP, anteroposterior; CT, computed tomography; CIRS, Computerized Imaging Reference Systems; IS, inferior‐superior; LR, left‐right. Source: Figure courtesy Sun Nuclear GmbH.

The CIRS Dynamic Thorax phantom 008A (Computerized Imaging Reference Systems (CIRS) Inc., Norfolk, VA) was used to simulate a specified breathing motion for both the lung in anteroposterior direction (AP), left‐right (LR), inferior‐superior direction (IS), and the surrogate platform in AP (Figure 2).^39^, ^40^ The phantom consists of anthropomorphic tissues such as tissue‐equivalent lung, soft tissue, cortical, and trabecular bones.

**FIGURE 3:**
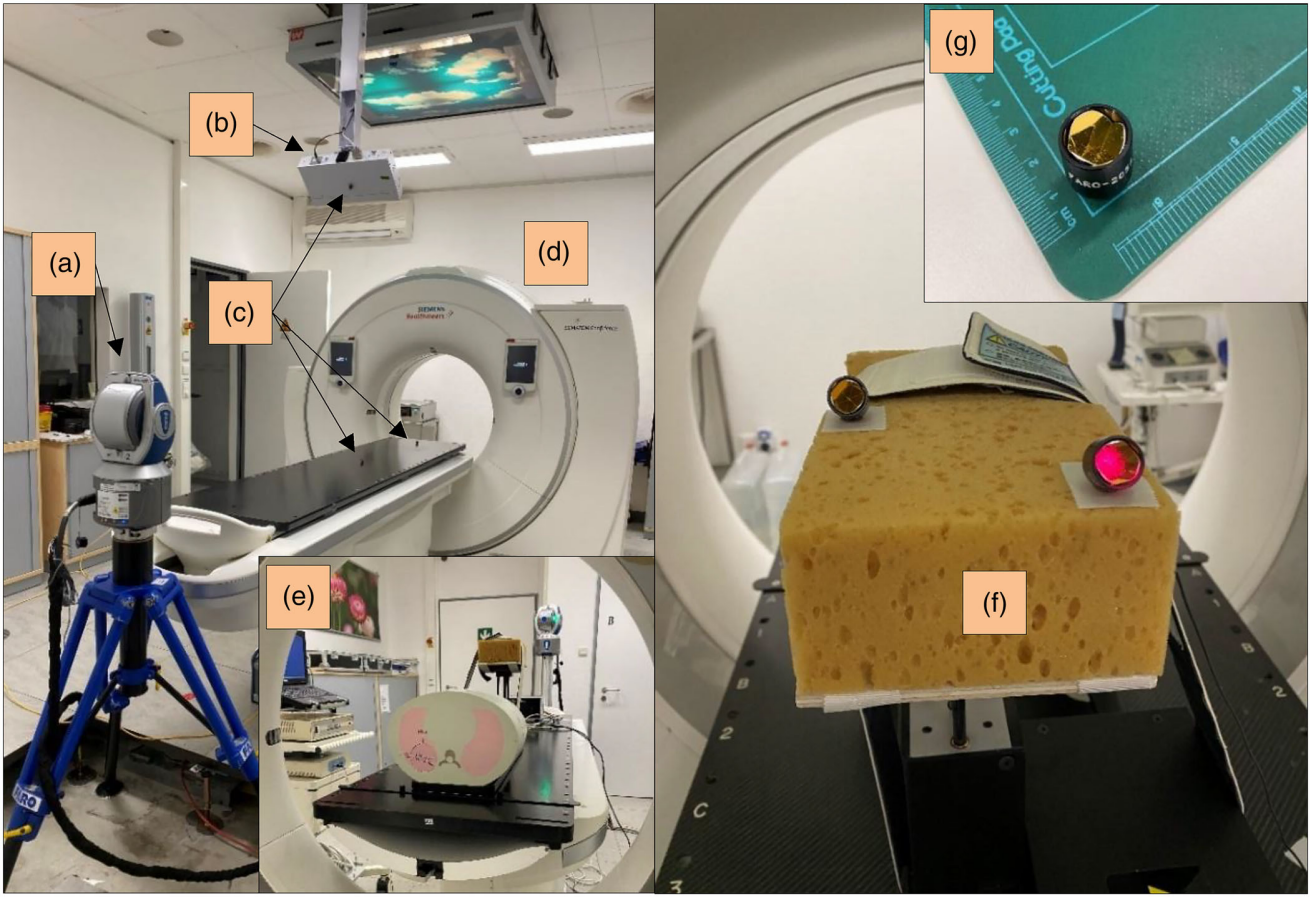
The setup of the 3D laser tracker in the CT scanner room (a) including the SimRT camera pod (b), the reflectors for the laser tracker (c), CT scanner (d), CIRS phantom setup with laser tracker (e), reflectors placed on the surrogate (f), and a Faro reflector (g). 3D, 3‐dimensional; CT, computed tomography.

This research used spherical tumor targets with 1, 2, and 3 cm diameters inside the lung insert. As a surrogate, either a brown sponge of 10 cm × 20 cm × 15 cm (Figure 3f) or a green Styrodur block of 15 cm× 20 cm × 10 cm (Figure 1h) were used and fixed on the rigid phantom platform. The sponge was used due to the requirement of an elastic surface as a surrogate for the pressure sensor to achieve a patient‐equivalent body surface.

### 2.4 Characteristics of simulated breathing patterns

Simulations of regular and irregular breathing patterns with different periods and peak‐to‐peak amplitudes were provided as the ground‐truth by the CIRS phantom (Table 2). Cos^4^ patterns were simulated in MATLAB 2022a (MathWorks, Natick, MA). The following Equation (1) describes the shape of the Cos breathing pattern:

(1) $$x\;\left(t\right)=\;A\cdot \cos{\left(\pi \cdot \frac{1}{T}\cdot t\right)}^{4}$$

**TABLE 2:** Characteristics of the used breathing patterns.

Breathing pattern	T ± SD (s)	A ± SD (mm)
**Cos*^4^*** **C1‐5** ^a^	4	2
	4	4
	4	8
	2	16
	4	16
**Volunteer with regular breathing pattern** **RV1‐4**	7.30 ± 0.52	10.43 ± 1.31
	6.66 ± 0.25	16.28 ± 1.23
	7.32 ± 0.33	17.02 ± 1.68
	6.31 ± 0.20	20.78 ± 1.54
**Volunteer with irregular breathing pattern** **IRV1‐4**	9.23 ± 3.60	13.33 ± 3.53
	6.74 ± 0.61	15.29 ± 2.01
	6.24 ± 2.04	14.42 ± 3.36
	6.45 ± 1.08	18.18 ± 1.93

Abbreviations: A, peak‐to‐peak amplitude; C, Cos^4^; CT, computed tomography; SD, standard deviation; T, period.

^a^ Measurements performed during and without CT scan respectively.

where x(t) is the breathing pattern at a given time t, A is the peak‐to‐peak amplitude, and $\frac{1}{T}$ the cosine wave frequency. Moreover, the breathing patterns of the volunteers were acquired using the Anzai system and then transferred to the CIRS phantom.

### 2.5 Quality assurance of SimRT

It was required to check that the pod position was not displaced relative to the last calibration based on the daily quality assurance (QA) before using SimRT. The monthly system calibration is performed at the isocenter of the CT bore using the SimRT calibration plate, which works as the plate of the AlignRT system^27^ where both plates differ in the shape. The isocenter point in the CT bore is determined through calibration and the breathing pattern is calculated relative to this point. In order to ensure that SimRT measures the breathing pattern in precisely the same longitudinal axis as the couch is moving and to make necessary adjustments to the 3D patient surface during the scan independently of the longitudinal direction of the couch, the calibration is also performed in an additional in‐bore position of 250 mm from the first isocenter point.

### 2.6 Data acquisition

In order to have a consistent comparison under the same conditions, the same experimental setup, as shown in Figures 1, 2, 3, was used including system settings of SimRT and Anzai, ambient light, mid‐skin tone, Anzai's pressure sensor “type high”, the same CT couch movement and CT scan region. The high‐pressure sensor was used due to its capability to measure deep breathing amplitudes.^7^ Furthermore, since Anzai measures pressure changes without a specific unit of measurement, the breathing patterns recorded by Anzai were initially normalized and then scaled by the given peak‐to‐peak amplitude value applied by the CIRS phantom, assuming that there is a linear relationship between the provided amplitude and the measured breathing signal. Thus, the signal linearity for both Anzai and SimRT was validated in prior of each measurement setup (Figure S1). This method of normalization and subsequent amplitude scaling for peaks and valleys was aimed to compare the breathing patterns quantitively. However, it's essential to note that for subsequent 4DCT reconstructions, the original, unaltered breathing patterns without the normalization and scaling process were exclusively used.

### 2.7 Reproducibility, spatial, and temporal accuracy of breathing patterns

The image quality of 4DCT reconstructions depend on the detection accuracy of the breathing patterns. Inaccurate breathing patterns can lead to distortions in tumor volume, shape, and trajectory in 4DCT images. Therefore, simultaneous measurements using SimRT and Anzai were performed with and without CT scans (Figure 1). A measurement without a CT scan means a measurement without the CT irradiation and couch movement. For this purpose, the SimRT patch was placed on one side of the surrogate (sponge), while the Anzai belt was placed on the opposite side. Regular and irregular breathing patterns were tracked for 60 and 120 s, respectively. As a benchmark for the comparisons, the measurement by both SimRT and Anzai was first initiated before running the surrogate movement. Then, the first real peak was considered a match point for analyzing both systems. Furthermore, the breathing patterns of SimRT were interpolated using the spline‐interpolation since the sampling rates of the two systems are different. In order to investigate the spatial and temporal accuracy of the breathing patterns, the precision of signal detection, including the signal period (T), detection of real peak time, tag peak time, real valley time, tag valley time, and nominal valley time, were analyzed. The tag value is the value determined by the software algorithm of Anzai or SimRT, and the real value is the value determined by our in‐house MATLAB tool. The following Equation (2) describes the calculation of the nominal valley time^41^:

(2) $$\begin{array}{c} \mathrm{Nominal}\;\mathrm{valley}\;\mathrm{tag}\left[i\right]=\frac{\mathrm{Peak}\;\mathrm{tag}\;\left[i+1\right]+\mathrm{Peak}\;\mathrm{tag}\;\left[i\right]}{2} \end{array}$$

where ith and ith + 1 are two consecutive tag peaks. In order to assess the reproducibility and similarity of the breathing patterns, the PC and MAD were ascertained. The MAD calculates the mean value of the absolute deviation of paired observations (ground‐truth and measurement). Mean values and standard deviations (SD) were calculated for all parameters in this work. The phase difference was calculated from the time shift of the peak and valley tag times.

### 2.8 Comparison of tumor trajectory, shape, and volume in 4DCTs

This experiment was performed to investigate the reliability and reconstruction accuracy of SimRT by tracking the surrogate surface (Figure 1h) and the tumor target inside the lung (Figure 1b–g). A periodic breathing pattern of cos^4^ was used for all tests, and the results were compared with Anzai, ground‐truth and static 3DCTs. Furthermore, the first test aimed to quantify the tracking accuracy of the surrogate. Thus, a Styrodur block was used as a surrogate with three imaging target inserts, each including a spherical tumor target with a 1, 2, and 3 cm diameter. The second test purposed to investigate the tumor shape, trajectory and volume in the obtained CT images. Therefore, the tumor target inside the lung was analyzed by using different peak‐to‐peak amplitudes: (i) trajectory A with AP = 16 mm, LR = 10 mm, IS = 10 mm and surrogate = 16 mm, and (ii) trajectory B with 2 mm for all motions. The parameters used during image acquisition are presented in Table 1.

The simultaneous acquisition using both SimRT and Anzai was not possible. Hence, the dynamic 4DCT scans were performed consecutively and divided into 10% phases (inhale and exhale) since it was assumed that this method had no impact on the reconstructed 4DCT images when using periodic breathing patterns. A uniform phantom region with the same scan length was acquired. Static 3DCT scans were acquired on six different breathing phases: 0% inhale, 50% inhale, 75% inhale, 100% inhale, 75% exhale, and 50% exhale. Using the Syngo.via software (VB60, Siemens Healthineers, Erlangen, Germany), the tumor target in each CT dataset of the different breathing phases was automatically segmented using a fixed Hounsfield unit threshold. The latter threshold was determined from the static 3DCT at 0% inhale as a reference to avoid intraobserver variations. The mid‐position of the target contours was tracked through the different 3DCTs (breathing phases), and the volume within the contours was evaluated. All scans were performed three times to calculate the mean and SD for all quantities in this work.

### 2.9 CT couch baseline drift

During previous experiments in Sections 2.7 and 2.8, a baseline drift of the breathing patterns during the CT scans was observed on the amplitude scale, when moving the CT couch in the longitudinal direction. Hence, measurements during the CT acquisition in six degrees of freedom using a 3D laser tracker (Faro Technologies Inc, Lake Mary, FL) were performed to determine the source for this drift. For this purpose, 3D markers were positioned on the CT couch, CIRS surrogate, and SimRT camera pod and tracked during the 4DCT scans (Figure 3).

## 3 RESULTS

### 3.1 Reproducibility, spatial, and temporal accuracy of breathing patterns

**FIGURE 4:**
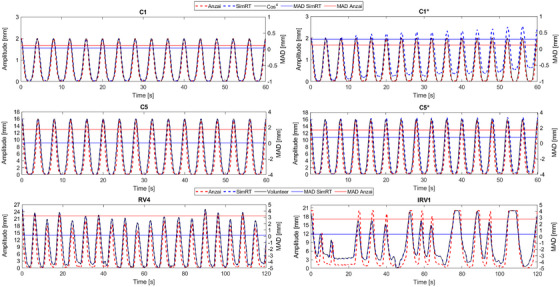
Six breathing patterns measured by Anzai and SimRT using the CIRS phantom compared with the ground‐truth. A = amplitude; C1 = cos^4^ (A = 2 mm, without CT); C1* = cos^4^ (A = 2 mm, during CT); C5 = cos^4^ (A = 16 mm, without CT); C5* = cos^4^ (A = 16 mm, during CT). CT, computed tomography; CIRS, Computerized Imaging Reference Systems; IRV, volunteer with irregular breathing pattern; MAD, mean absolute deviation; RV, volunteer with regular breathing pattern.

Figure 4 and Figure S2 depict the ground‐truth and measurements of regular and irregular breathing patterns (RV and IRV, respectively) for Anzai and SimRT without and during the CT scans. Table 3 shows the measured peak‐to‐peak amplitudes, MAD, and PC values for all breathing patterns.

**TABLE 3:** Summary of real, tag peak‐to‐peak amplitudes and MADs between the ground‐truth and measurements recorded by SimRT and Anzai for all regular and irregular breathing patterns.

	SimRT				Anzai			
	Mean real‐value	Mean tag‐value			Mean real‐value	Mean tag‐value		
Breathing pattern	A ± SD [mm]	A ± SD [mm]	MAD ± SD [mm]	PC	A ± SD [mm]	A ± SD [mm]	MAD ± SD [mm]	PC
C1	1.98 ± 0.02	1.96 ± 0.02	0.04 ± 0.02	0.998	1.96 ± 0.02	1.94 ± 0.01	0.11 ± 0.10	0.987
C2	3.90 ± 0.01	3.88 ± 0.01	0.12 ± 0.08	1.000	3.54 ± 0.00	3.51 ± 0.06	0.27 ± 0.28	0.985
C3	8.00 ± 0.01	7.99 ± 0.02	0.06 ± 0.04	1.000	7.88 ± 0.04	7.86 ± 0.03	0.58 ± 0.55	0.982
C4	15.88 ± 0.03	15.85 ± 0.05	0.37 ± 0.27	1.000	15.98 ± 0.05	15.90 ± 0.11	1.29 ± 1.11	0.980
C5	16.04 ± 0.02	16.02 ± 0.02	0.05 ± 0.04	1.000	15.60 ± 0.12	15.52 ± 0.09	1.75 ± 1.54	0.965
C1*	2.01 ± 0.03	1.99 ± 0.03	0.30 ± 0.18	0.975	2.00 ± 0.00	1.99 ± 0.02	0.12 ± 0.11	0.987
C2*	4.01 ± 0.03	3.98 ± 0.04	0.34 ± 0.24	0.991	3.94 ± 0.02	3.92 ± 0.02	0.18 ± 0.16	0.994
C3*	8.08 ± 0.03	8.04 ± 0.05	0.45 ± 0.31	0.999	7.79 ± 0.05	7.78 ± 0.05	0.62 ± 0.55	0.984
C4*	16.02 ± 0.05	16.02 ± 0.05	1.23 ± 0.87	0.999	15.87 ± 0.04	15.83 ± 0.05	1.81 ± 1.87	0.910
C5*	16.07 ± 0.03	16.06 ± 0.05	0.80 ± 0.53	0.999	15.66 ± 0.07	15.63 ± 0.07	1.72 ± 1.53	0.965
RV1	9.83 ± 1.07	9.60 ± 1.04	0.35 ± 0.38	0.991	13.02 ± 2.37	12.83 ± 2.34	3.63 ± 1.43	0.948
RV2	16.21 ± 1.25	15.79 ± 1.36	0.40 ± 0.36	0.996	13.00 ± 2.59	12.97 ± 2.56	5.85 ± 3.61	0.791
RV3	16.91 ± 1.67	16.84 ± 1.69	0.55 ± 0.49	0.993	16.79 ± 2.47	16.63 ± 2.47	3.03 ± 2.17	0.933
RV4	20.48 ± 1.49	20.20 ± 1.47	0.18 ± 0.17	0.998	18.80 ± 2.38	18.70 ± 2.30	3.22 ± 2.29	0.861
IRV1	13.18 ± 3.66	12.91 ± 3.52	0.36 ± 0.36	0.996	16.65 ± 3.99	16.54 ± 3.96	2.76 ± 2.02	0.907
IRV2	15.00 ± 2.10	14.21 ± 1.98	0.27 ± 0.17	0.998	11.88 ± 4.62	11.60 ± 4.63	2.96 ± 2.56	0.861
IRV3	14.49 ± 2.70	14.07 ± 2.97	0.92 ± 1.02	0.968	18.30 ± 4.37	18.23 ± 4.36	3.24 ± 2.55	0.831
IRV4	17.16 ± 2.21	15.95 ± 2.91	0.23 ± 0.15	0.999	16.41 ± 6.75	16.39 ± 6.78	5.04 ± 1.72	0.970

*Note*: First 10 periods were considered for the evaluation.

Abbreviations: A, amplitude; CT, computed tomography; C, cos^4^ without CT; C*, cos^4^ during CT; IRV, volunteer with irregular breathing pattern; MAD, mean absolute deviation; PC, Pearson correlation; RV, volunteer with regular breathing pattern; SD, standard deviation.

^a^ Measurements during the CT scan.

By comparison with ground‐truth, SimRT showed maximal amplitude differences of 0.12 and 0.15 mm for real and tag amplitudes without CT scan, respectively, while 0.08 and 0.06 mm for real and tag amplitudes were observed during CT scan, respectively, when using cos^4^ (Table 3). For RV breathing patterns, SimRT displayed higher amplitude differences of 0.60 and 0.83 mm for real and tag amplitudes, respectively, while 1.02 and 2.23 mm for real and tag amplitudes were observed, respectively, when using IRV patterns (Table 3). Concerning SimRT, Anzai showed greater maximal differences for real and tag amplitudes compared to ground‐truth when using cos^4^ (except for C1*), but less than 1 mm, while more significant differences up to 3.31 and 3.88 mm for RV and IRV patterns were provided, respectively. Furthermore, it was noticed that the tag amplitudes were smaller than the real amplitudes in most measurements but within 1 mm for both systems (Table 3).

In addition, the breathing patterns of the volunteers and cos^4^ captured by SimRT were reproduced with a maximal MAD (± SD) of 0.37 ± 0.27, 1.23 ± 0.87, 0.55 ± 0.49, and 0.92 ± 1.02 mm for C, C*, RV and IRV patterns, respectively, whilst higher MADs of 1.75 ± 1.54, 1.81 ± 1.87, 5.85 ± 3.61, and 5.04 ± 1.72 mm were showed in Anzai for C, C*, RV and IRV patterns, respectively (Table 3). Table 3 also displays a higher PC range for SimRT than Anzai, between 0.968 for the IRV3 pattern and 1 for C2‐5. Anzai showed a maximal PC range between 0.791 for the RV2 pattern and 0.994 for C2*. Due to the CT couch baseline drift, SimRT showed a weaker correlation for measurements during CT scans. Figure 4 and Figure S2 confirm the strong correlation of SimRT with the ground‐truth. Additionally, volunteer breathing patterns showed smaller correlations than cos^4^ patterns for both systems. Moreover, our results showed that Anzai has a different shape of breathing curve than the ground‐truth and SimRT (narrower breathing patterns).

Further, Table 4 shows that the peak recognition in SimRT was more precise and reproducible with a maximum mean (± SD) of 40 ± 40, 30 ± 30, 30 ± 40, and 100 ± 10 ms, in comparison to Anzai with 150 ± 0, 110 ± 30, 120 ± 20, and 160 ± 80 ms for C, C*, RV, and IRV patterns, respectively. The periods of cos^4^ breathing patterns were more accurately reproduced with a maximum SD of 20 and 30 ms for SimRT and Anzai, respectively, whereas the periods of volunteer patterns were less accurate with a maximum mean of 30 and 2080 ms, respectively. Moreover, the Anzai's valley detection's precision was less accurate than SimRT. Where Anzai located the tag valley in 94% before the nominal position with a precision between 47 ± 20 and 1710 ± 34 ms, SimRT placed them in 83% after the nominal valley position and showed a more precise valley recognition between 30 ± 50 and 100 ± 190 ms (Table 4).

**TABLE 4:** Precision of tagging for peaks and valleys showing the time difference between real and tag values determined by the SimRT and Anzai algorithms for regular and irregular breathing patterns, respectively.

	SimRT			Anzai		
	Mean ± SD (s)			Mean ± SD (s)		
Breathing pattern	Period	∆T (real peak—tag peak)	∆T valley	Period	∆T (real peak—tag peak)	∆T valley
C1	4.00 ± 0.02	0.04 ± 0.04	0.01 ± 0.01	4.00 ± 0.00	0.06 ± 0.01	‐0.47 ± 0.02
C2	4.00 ± 0.02	0.03 ± 0.03	0.00 ± 0.01	4.00 ± 0.00	0.05 ± 0.03	‐0.30 ± 0.00
C3	4.00 ± 0.02	0.02 ± 0.02	0.00 ± 0.01	4.00 ± 0.00	0.03 ± 0.01	‐0.29 ± 0.01
C4	2.00 ± 0.01	0.00 ± 0.00	0.03 ± 0.05	2.00 ± 0.00	0.15 ± 0.00	‐0.05 ± 0.00
C5	4.00 ± 0.02	0.02 ± 0.02	0.00 ± 0.01	4.00 ± 0.03	0.03 ± 0.02	‐0.29 ± 0.02
C1*	4.00 ± 0.02	0.03 ± 0.03	0.10 ± 0.19	4.00 ± 0.00	0.08 ± 0.04	‐0.40 ± 0.00
C2*	4.00 ± 0.02	0.02 ± 0.02	0.00 ± 0.01	4.00 ± 0.00	0.11 ± 0.03	‐0.65 ± 0.00
C3*	4.00 ± 0.02	0.02 ± 0.02	0.00 ± 0.01	4.00 ± 0.00	0.03 ± 0.02	‐0.30 ± 0.01
C4*	2.00 ± 0.02	0.02 ± 0.02	0.01 ± 0.01	2.00 ± 0.00	0.00 ± 0.01	‐0.08 ± 0.01
C5*	4.00 ± 0.02	0.02 ± 0.02	‐0.10 ± 0.01	4.00 ± 0.00	0.05 ± 0.02	‐0.34 ± 0.01
RV1	7.31 ± 0.53	0.03 ± 0.04	0.00 ± 0.01	7.32 ± 0.52	0.08 ± 0.01	‐0.57 ± 0.35
RV2	6.66 ± 0.31	‐0.01 ± 0.07	0.00 ± 0.01	6.67 ± 0.17	0.02 ± 0.16	‐1.71 ± 0.34
RV3	7.35 ± 0.33	‐0.02 ± 0.04	0.01 ± 0.01	7.34 ± 0.31	0.12 ± 0.02	‐0.69 ± 0.37
RV4	6.32 ± 0.20	0.02 ± 0.04	‐0.10 ± 0.01	6.31 ± 0.20	0.08 ± 0.03	‐0.63 ± 0.16
IRV1	9.21 ± 3.59	‐0.07 ± 0.09	0.00 ± 0.01	9.13 ± 3.35	0.16 ± 0.08	‐1.46 ± 2.07
IRV2	6.74 ± 0.67	0.03 ± 0.08	‐0.10 ± 0.01	4.66 ± 2.11	0.06 ± 0.05	‐0.45 ± 0.57
IRV3	6.25 ± 2.10	0.04 ± 0.07	0.00 ± 0.02	6.26 ± 2.07	0.06 ± 0.06	‐0.98 ± 1.15
IRV4	6.49 ± 1.16	0.10 ± 0.01	0.01 ± 0.01	5.04 ± 1.92	0.06 ± 0.03	‐0.38 ± 0.58

*Note*: The difference between the tag nominal valley and tag valley values were calculated. For the period, the difference between the tag peak values and the first 10 periods were considered for the calculation.

Abbreviations: C, cos^4^ without CT; C*, cos^4^ during CT; CT, computed tomography; IRV, volunteer with irregular breathing pattern; RV, volunteer with regular breathing pattern; SD, standard deviation; ∆T, time difference.

^a^ Measurements during the CT scan.

On the contrary to the Anzai stable sampling rate of 40 Hz, the sampling rate of SimRT slightly varies during one measurement with 39.34 ± 1.5 Hz on the Siemens CT scanner. The histogram (Figure S3) illustrates all sampling rates recorded during one measurement. Similar sampling rates were observed for all measurements, including regular and irregular breathing patterns without and during the CT scan.

### 3.2 Comparison of tumor trajectory, shape, and volume in 4DCTs

**FIGURE 5:**
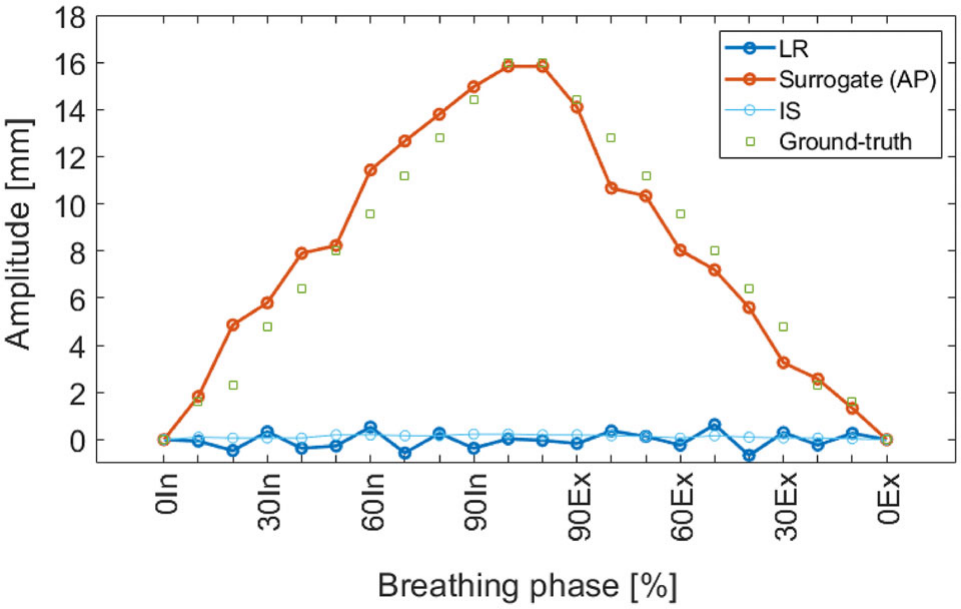
Tracking performance of SimRT illustrating the surrogate tracking in AP using a 16 mm peak‐to‐peak amplitude. Maximal SD was ±0.29 mm in AP for 75% exhale. A Styrodur was used here as a surrogate. AP, anteroposterior; Ex, exhale; IS, inferior‐superior; In, inhale; LR, left‐right.

Figure 5 shows the accuracy of SimRT by tracking and quantifying the surrogate movement by sorting the different inhale and exhale phases of a 4DCT dataset to reproduce the given breathing pattern of cos^4^. Compared to the given ground‐truth, the mean variation along all breathing phases was −0.06 ± 1.16 mm with a maximum of −2.57 mm in 20% inhale. LR and IS amplitudes showed negligible movements during the breathing process.

**FIGURE 6:**
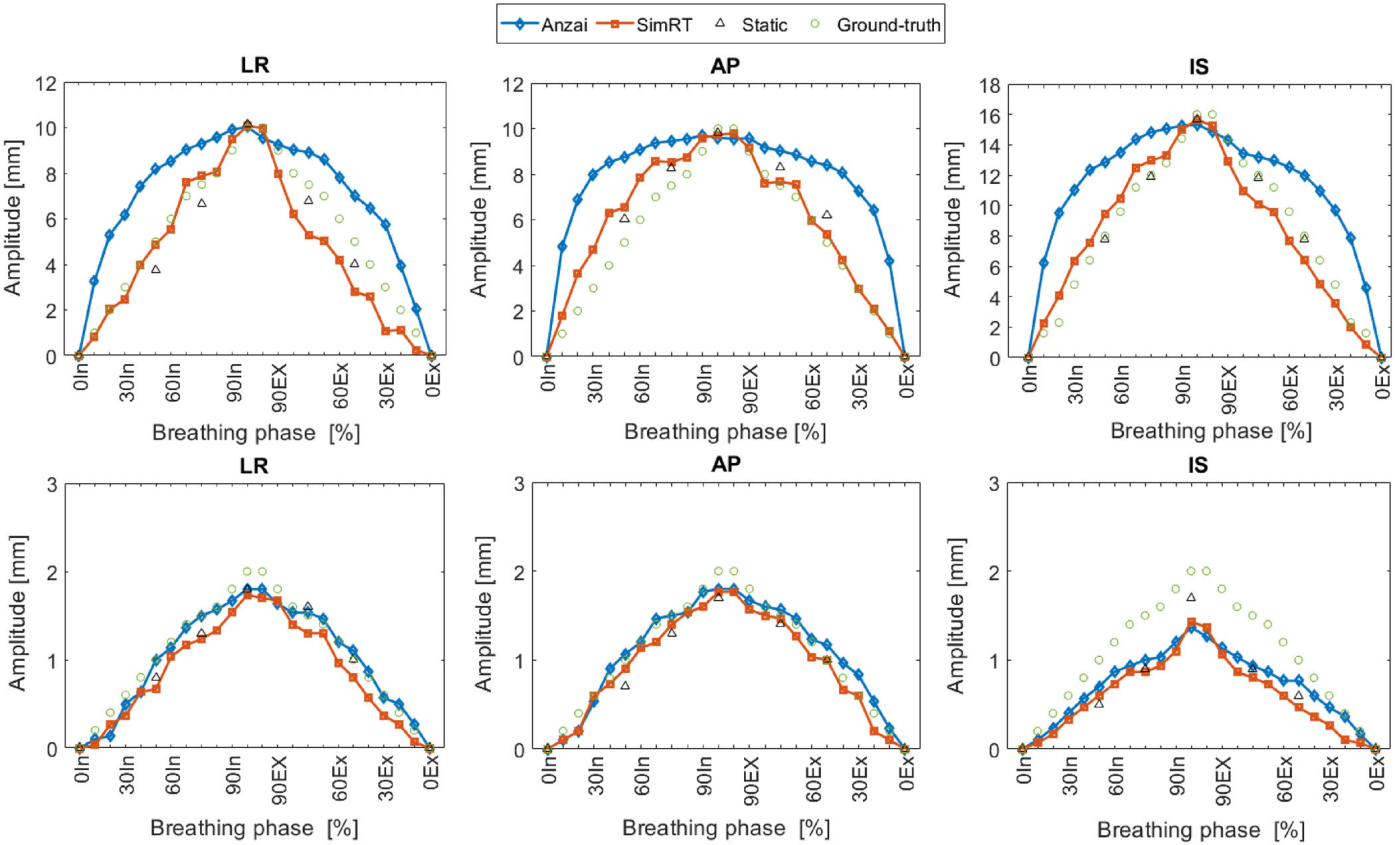
Tumor trajectory inside the CIRS phantom in LR, IS, and AP directions using 10, 16, and 10 mm for the first experiment A (upper three diagrams) and 2 mm for the second B (lower three diagrams). Maximal SD was ±1.4 mm in AP, ±0.7 mm in IS, and ±0.3 mm in IS for Anzai, SimRT and Static, respectively. A sponge was used here as a surrogate. AP, anteroposterior; Ex, exhale; IS, inferior‐superior; In, inhale; Static, static 3DCT scan; LR, left‐right; SD, standard deviation.

Figure 6 depicts the tumor trajectories (A and B) obtained from Anzai and SimRT versus ground‐truth and static 3DCT. The upper trajectories A obtained by Anzai reveal clearly higher differences from ground‐truth with a maximum of −3.4, −7.2, and −5 mm in LR, IS, and AP, respectively, while SimRT shows considerably fewer deviations with a maximum of 2.2, 1.9, and −2.3 mm in LR, IS, and AP, respectively. For the lower trajectories B, both systems Anzai and SimRT provide similar deviations from ground‐truth with 0.3, 0.7, and 0.2 mm in LR, IS, and AP, respectively. By contrast with the ground‐truth, the static 3DCTs agrees with SimRT and Anzai when using trajectory B, while noticeably smaller maximum differences <1.3 mm are applied when using trajectory A. Note that all differences are relative to 0% inhale and negative deviations mean that the system provides a larger trajectory difference from the expected ground‐truth.

Table 5 exhibits axial images of the tumor mid‐position in six selected breathing phases of tumor trajectory A, reconstructed using SimRT and Anzai compared to static 3DCT images. The volumetric discrepancies of the tumor between SimRT, Anzai, static 3DCT, and ground‐truth are presented in details in Figure S4 (using boxplots), Tables S1 and S2. The mean volume deviations of static 3DCT, Anzai, and SimRT from ground‐truth were −0.42 ± 98%, 1.71 ± 1.62%, and 1.24 ± 2.02% for trajectory A, respectively. In comparison, −0.13 ± 1.95%, 0.20 ± 1.75%, and 0.08 ± 1.48% were ascertained for trajectory B, respectively.

**TABLE 5:** Axial mid‐position sections of the 4DCT images reconstructed by using breathing patterns from both Anzai and SimRT compared to the static 3DCT.

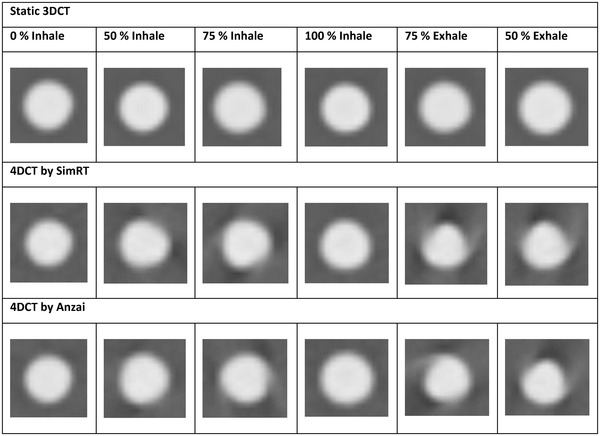

*Note*: A tumor target with a diameter of 1 cm was used. This table represents the upper tumor trajectory A depicted in Figure 6. Abbreviations: CT, computed tomography; 3D, 3‐dimensional; 4D, 4‐dimensional.

### 3.3 CT couch baseline drift

The obtained results showed that the couch drifts, when moving into or out of the CT‐bore, are the source of the baseline shift registered by SimRT. This drift, presented previously in Figure 4 and Figure S2 (C1*‐C5*), depends on the weight. The maximal translational deviations while moving the CT couch with 80 Kg for a 1.5 m range into the CT‐bore are 0.7 mm lateral, 4 mm longitudinal, and 14 mm vertical. The maximal rotational deviations were 0.02° in yaw, 0.05° in roll, and 0.5° in pitch. The camera pod of SimRT did not show any considerable changes in position. Thus, both translational and rotational deviations were negligible.

Another experiment with a CT scan of 45 cm out of the CT‐bore using the CIRS phantom with a weight of 20 Kg showed that the CT couch drifted about 1.4 mm vertically. This latter drift was detected in both the laser tracker using a reflector on the surrogate and SimRT. Additionally, results confirmed that this drift did not affect the peak‐to‐peak amplitudes of the investigated breathing patterns.

## 4 DISCUSSIONS

### 4.1 Reproducibility, spatial, and temporal accuracy of the breathing patterns

The reproducibility, spatial, and temporal accuracy of regular and irregular breathing patterns were investigated and compared using different external RMSs in previous studies (such as optical, mechanical and spirometric devices).^6^, ^8^, ^22^, ^26^, ^41^, ^43^ The results of our work (Table 3) showed that SimRT provided a more accurate, stable and consistent peak‐to‐peak amplitude detection than Anzai. Anzai may be affected by the physical belt setup and point‐based pressure sensor. However, both systems exhibited a good peak‐to‐peak amplitude tagging within 1 mm compared to measured real amplitudes. The MAD values presented in this study showed the relation between both systems' breathing reproducibility and detection accuracy. Compared to ground‐truth, SimRT more precisely reproduced the breathing signals with a MAD range <1 mm versus Anzai with a 1–6 mm range for regular and irregular breathing patterns, respectively. In reality, patients breathe irregularly and non‐reproducibly, which can lead to increased MAD values compared to phantom measurements.

Moreover, it was found that Anzai provides narrower shaped breathing patterns compared to SimRT and ground‐truth, leading to phase differences in recorded breathing information, especially when using irregular breathing patterns (Figure 4 and Figure S2). The inaccurate tag valley detection may cause this phase difference of Anzai since Anzai records pressure variations of the tidal body volume, which disappear in the end‐expiration phase, that is, no pressure. Such a phase shift is a known problem for point‐based RMSs such as Anzai.^8^, ^22^

Furthermore, SimRT exhibited a stronger correlation with ground truth across all measurements (Table 3), including small amplitudes (e.g., 2 mm), compared to Anzai. Kauweloa et al.^6^ investigated the surface‐guided GateCT (VisionRT Ltd, London, United Kingdom) versus the real‐time position management, RPM system (Varian, Palo Alto, CA). In contrast to their work, the surface‐guided SimRT system confirmed its accuracy in both phase and amplitude tracking.

In addition, SimRT showed a more accurate peak and valley recognition in all measurements versus Anzai, whereas Anzai's peak recognition was more precise than the valley recognition (Table 4). The predictive algorithm used in both systems may also affect peak and valley tagging variation. A C Vásquez et al.^41^ confirmed Anzai's phase difference and worse valley recognition compared to the GateCT system.

Nevertheless, it is essential to highlight the necessity of validating linearity before implementing the normalization and subsequent amplitude scaling method. Moreover, it's important to clarify that this method is exclusively intended for use in phantom studies. Ensuring signal linearity in RMSs is essential to prevent any additional distortion from affecting the correlation between tumor mobility and the recorded breathing signal.^7^

Since the sampling rate of SimRT varies during one measurement (Figure S3), inaccurate frequencies may lead to incorrect peak and valley detection. For this reason, the phase‐ and amplitude‐based sorting algorithms can be affected, and incorrect re‐sorting of the CT projections may produce image artifacts, leading to incorrect ITV estimations. Consequently, the quality of radiotherapy (gated and nongated) can be compromised. Despite these variations in the sampling rate, the temporal and spatial accuracy of SimRT are still more precise and consistently reproduced versus Anzai, especially by irregularities in breathing patterns based on phantom measurements.

### 4.2 Comparison of tumor trajectory, shape, and volume in 4DCTs

The greater the precision of the recorded breathing pattern, the more accurate the reconstructed ITV will be. For both tumor trajectories (A and B) examined under complex conditions involving movement in AP, LR, and IS directions simultaneously (Figure 6), SimRT yielded a more accurate tumor localization, exhibiting a mid‐position deviation ranging from 12% to 23%. In contrast, Anzai demonstrated 34% to 50% deviations compared to the ground truth. Furthermore, our investigation revealed that as the amplitude increases, the deviation also rises for both systems. The greater deviations observed when employing the Anzai system are attributed to phase differences, motion effects, and partial volume artifacts. However, SimRT was primarily influenced by motion and partial volume artifacts. The presence of the latter artifacts in 4DCT images has been explained by Watkins et al.,^44^ Rietzel et al.,^45^ and Nakamura et al.^46^

Exhibiting a maximum deviation of 16% (in 20% inhale), SimRT demonstrated an accurate quantification of surrogate movement in comparison to ground truth (Figure 5). Compared to end‐exhalation and ‐inhalation, deviations up to 1% were achieved. That being said, reducing target velocity leads to reduced motion artifacts and more accurate target position.

Moreover, it is important to consider the interplay arising between the tumor motion in various directions (AP, LR, and IS) and the motion of the CT couch and scanner during the image acquisition, as Lewis et al. reported in their work.^47^ The originally spherical tumor shape exhibited significant distortions, particularly during breathing phases characterized by a higher motion velocity, such as 75% inhale/exhale, where motion artifacts are more pronounced (Table 5).

The volume discrepancies among SimRT, Anzai, and the ground truth exhibited similarities for both trajectories (Figure S4, Tables S1–S2). However, it appears that peak‐to‐peak amplitude influences the volume discrepancy when comparing both trajectories, A and B. In comparison to the static 3DCT scan, the volume discrepancy was less impacted by the amplitude. These alterations in tumor volume are ascribed to the effects of motion and partial volume artifacts, which can potentially affect the target shape and contouring precision within the treatment planning system.

### 4.3 CT couch baseline drift

Despite the CT baseline drift, the peak‐to‐peak amplitude, period values, valley, and peak recognition with and without CT scans, presented in Tables 3 and  4, provided similar deviations from ground‐truth. That being said, the temporal and spatial accuracy were not affected during the CT couch movement and an error in the phase‐based sorting algorithm of our CT scanner can be excluded.

A C Vásquez et al.^41^ supposed that the baseline drift of GateCT may cause a delay in the valley detection and Kauweloa et al.^6^ cautioned its use with amplitude‐based sorting algorithm. On the contrary, our results support the use of SimRT with amplitude‐based sorting approaches. Nevertheless, SimRT should be investigated on a CT scanner with an amplitude‐based sorting algorithm.

Moreover, Schick et al.^26^ reported a non‐defined baseline drift of ≤ ±2 mm to be considered by using the Varian respiratory gating system for CT scanners (RGSC) on the Brilliance CT Big Bore scanner (Philips Healthcare, Amsterdam, Netherlands), when the camera system is mounted on the wall or the ceiling, which corresponds to the same CT couch baseline drift we found in our study. Furthermore, Heinz et al.^7^ reported a CT couch baseline drift on a Toshiba CT scanner (Toshiba Medical System Group, Tokyo, Japan), dependent on the scanner load.

### 4.4 Limitations and considerations

During the evaluation of SimRT, some limitations were noticed, such as (i) limited FOV (occlusion by immobilization devices and CT scanner), (ii) self‐occlusion by patients, (iii) retrospective import of the breathing pattern without a security query on the CT console, and (iv) patient body dependencies (e.g., skin tone, body hair). However, it is recommended prior to the 4DCT scan to ensure that a sufficient skin surface in the entire scan region is visible, the correct skin tone is configured in the SimRT software, the same ambient lighting is used as during the monthly QA and a noise‐free breathing pattern is available. These measures can help avoid patient re‐imaging, which can cause workflow delay and an additional dose contribution (if additional x‐ray imaging is used). In addition, it should be considered that our findings are based on phantom measurements using a flat surrogate surface and reproducible breathing patterns on the Somatom Confidence CT scanner. These findings could differ by using skin surfaces with geometrical variations or non‐reproducible breathing characteristics or on other CT scanners. Thus, results from phantom studies may not be directly transferable clinically.

## 5 CONCLUSIONS

This work aimed to validate the performance of the surface‐guided SimRT system compared to the Anzai pressure sensor before implementing it into the clinical workflow. For this purpose, the reconstruction, temporal and spatial accuracy were assessed using regular, irregular breathing patterns and a commercial anthropomorphic phantom on a Siemens CT scanner. In contrast to Anzai, SimRT showed a more accurate and stable breathing tracking, independent of the breathing pattern, amplitude and period, thus resulting in a more consistent temporal and spatial accuracy. These results lead to a more realistic (i.e., closer to the ground‐truth) breathing‐adapted treatment planning. Furthermore, SimRT can be used for both phase‐ and amplitude‐based 4DCT reconstructions. Nevertheless, instances of motion and partial volume artefacts are still in the reconstructed tumor target. These artifacts are more noticeable during breathing phases with greater target motion velocity when compared to the end‐breathing phases, such as 0% and 100% inhale.

In addition, system limitations should be investigated before using the system on patients since highly pronounced irregularities in patient breathing patterns may limit the reconstruction accuracy achieved in our phantom investigations. Finally, we recommend using same RMSs for both 4DCT‐based treatment planning and gated‐induced irradiation, since temporal and spatial inaccuracies would be applied to the 4DCT reconstructions by using different RMSs in RT, according to this research.

## CONFLICT OF INTEREST STATEMENT

The authors declare no conflicts of interest.

## Supporting information

Supporting Information

## Data Availability

Research data are stored in an institutional repository and will be shared upon request to the corresponding author.
