# Supplementary material for: Comparative evaluation of a surface‐based respiratory monitoring system against a pressure sensor for 4DCT image reconstruction in phantoms
Source: J Appl Clin Med Phys. 2023 Oct 10;25(2):e14174. doi: 10.1002/acm2.14174 (PMC10860430; doi:10.1002/acm2.14174)
Supplement: Supplementary file 1 — Supporting Information [file ACM2-25-e14174-s001.docx]

Table S1. Tumor volume and volume discrepancy (1-(Anzai/ground-truth), 1-(SimRT/ground-truth)) in different reconstructed 4DCT breathing phases. Note that the percent difference is calculated from the raw values after rounding to significant figures. Mean and SD are rounded to three decimal places for volume values and to two decimal places for volume deviations.

|  | Volume [cm^3^] | | | Volume deviation [%] | | |
| --- | --- | --- | --- | --- | --- | --- |
| Breathing phase [%] | **Static** | **Anzai** | **SimRT** | **Static vs. Ground-truth** | **Anzai vs. Ground-truth** | **SimRT vs. Ground-truth** |
| 0In | 0.520 | 0.520 | 0.528 | 0.00 | 0.00 | -1.54 |
| 10In |  | 0.513 | 0.523 |  | 1.35 | -0.58 |
| 20In |  | 0.513 | 0.503 |  | 1.35 | 3.27 |
| 30In |  | 0.500 | 0.528 |  | 3.85 | -1.54 |
| 40In |  | 0.503 | 0.505 |  | 3.27 | 2.88 |
| 50In | 0.515 | 0.518 | 0.510 | 0.96 | 0.38 | 1.92 |
| 60In |  | 0.513 | 0.518 |  | 1.35 | 0.38 |
| 70In |  | 0.523 | 0.498 |  | -0.58 | 4.23 |
| 75In | 0.523 | 0.505 | 0.505 | -0.58 | 2.88 | 2.88 |
| 80In |  | 0.510 | 0.498 |  | 1.92 | 4.23 |
| 90In |  | 0.518 | 0.525 |  | 0.38 | -0.96 |
| 100In | 0.520 | 0.505 | 0.520 | 0.00 | 2.88 | 0.00 |
| 100Ex |  | 0.485 | 0.523 |  | 6.73 | -0.58 |
| 90Ex |  | 0.503 | 0.505 |  | 3.27 | 2.88 |
| 80Ex |  | 0.515 | 0.508 |  | 0.96 | 2.31 |
| 75Ex | 0.530 | 0.518 | 0.520 | -1.92 | 0.38 | 0.00 |
| 70Ex |  | 0.513 | 0.503 |  | 1.35 | 3.27 |
| 60Ex |  | 0.515 | 0.515 |  | 0.96 | 0.96 |
| 50Ex | 0.525 | 0.508 | 0.503 | -0.96 | 2.31 | 3.27 |
| 40Ex |  | 0.510 | 0.508 |  | 1.92 | 2.31 |
| 30Ex |  | 0.510 | 0.510 |  | 1.92 | 1.92 |
| 20Ex |  | 0.520 | 0.523 |  | 0.00 | -0.58 |
| 10Ex |  | 0.518 | 0.533 |  | 0.38 | -2.50 |
| Mean | 0.522 | 0.511 | 0.514 | -0.42 | 1.71 | 1.24 |
| SD | 0.005 | 0.008 | 0.010 | 0.98 | 1.62 | 2.02 |

Tumor motion: AP = 16 mm, LR = 10 mm, IS = 10 mm. The volume of the ground-truth is 0.52 cm^3^. Abbreviations: AP = anteroposterior; CT = computed tomography; Ex = exhale; IS = inferior-superior; In = inhale; LR = left-right; Static = static 3DCT scan; SD = Standard deviation; 3D = 3-dimensional; 4D = 4-dimensional.

Table S2. Tumor volume and volume discrepancy (1-(Anzai/ground-truth), 1-(SimRT/ground-truth)) in different reconstructed 4DCT breathing phases. Note that the percent difference is calculated from the raw values after rounding to significant figures. Mean and SD are rounded to three decimal places for volume values and to two decimal places for volume deviations.

|  | Volume [cm^3^] | | | Volume deviation [%] | | |
| --- | --- | --- | --- | --- | --- | --- |
| Breathing phase [%] | **Static** | **Anzai** | **SimRT** | **Static vs. Ground-truth** | **Anzai vs. Ground-truth** | **SimRT vs. Ground-truth** |
| 0In | 0.530 | 0.520 | 0.533 | -1.92 | 0.00 | -2.50 |
| 10In |  | 0.503 | 0.507 |  | 3.27 | 2.50 |
| 20In |  | 0.517 | 0.510 |  | 0.58 | 1.92 |
| 30In |  | 0.517 | 0.520 |  | 0.58 | 0.00 |
| 40In |  | 0.517 | 0.517 |  | 0.58 | 0.58 |
| 50In | 0.520 | 0.527 | 0.520 | 0.00 | -1.35 | 0.00 |
| 60In |  | 0.530 | 0.530 |  | -1.92 | -1.92 |
| 70In |  | 0.533 | 0.530 |  | -2.50 | -1.92 |
| 75In | 0.507 | 0.537 | 0.517 | 2.50 | -3.27 | 0.58 |
| 80In |  | 0.520 | 0.520 |  | 0.00 | 0.00 |
| 90In |  | 0.523 | 0.520 |  | -0.58 | 0.00 |
| 100In | 0.527 | 0.513 | 0.513 | -1.35 | 1.35 | 1.35 |
| 100Ex |  | 0.513 | 0.517 |  | 1.35 | 0.58 |
| 90Ex |  | 0.520 | 0.527 |  | 0.00 | -1.35 |
| 80Ex |  | 0.513 | 0.517 |  | 1.35 | 0.58 |
| 75Ex | 0.510 | 0.527 | 0.533 | 1.92 | -1.35 | -2.50 |
| 70Ex |  | 0.533 | 0.523 |  | -2.50 | -0.58 |
| 60Ex |  | 0.510 | 0.520 |  | 1.92 | 0.00 |
| 50Ex | 0.530 | 0.507 | 0.510 | -1.92 | 2.50 | 1.92 |
| 40Ex |  | 0.513 | 0.510 |  | 1.35 | 1.92 |
| 30Ex |  | 0.507 | 0.510 |  | 2.50 | 1.92 |
| 20Ex |  | 0.523 | 0.520 |  | -0.58 | 0.00 |
| 10Ex |  | 0.513 | 0.527 |  | 1.35 | -1.35 |
| Mean | 0.521 | 0.519 | 0.520 | -0.13 | 0.20 | 0.08 |
| SD | 0.010 | 0.009 | 0.008 | 1.95 | 1.75 | 1.48 |

Tumor motion: AP = 2 mm, LR = 2 mm, IS = 2 mm. The volume of the ground-truth is 0.52 cm^3^. Abbreviations: AP = anteroposterior; CT = computed tomography; Ex = exhale; IS = inferior-superior; In = inhale; LR = left-right; Static = static 3DCT scan; SD = Standard deviation; 3D = 3-dimensional; 4D = 4-dimensional.


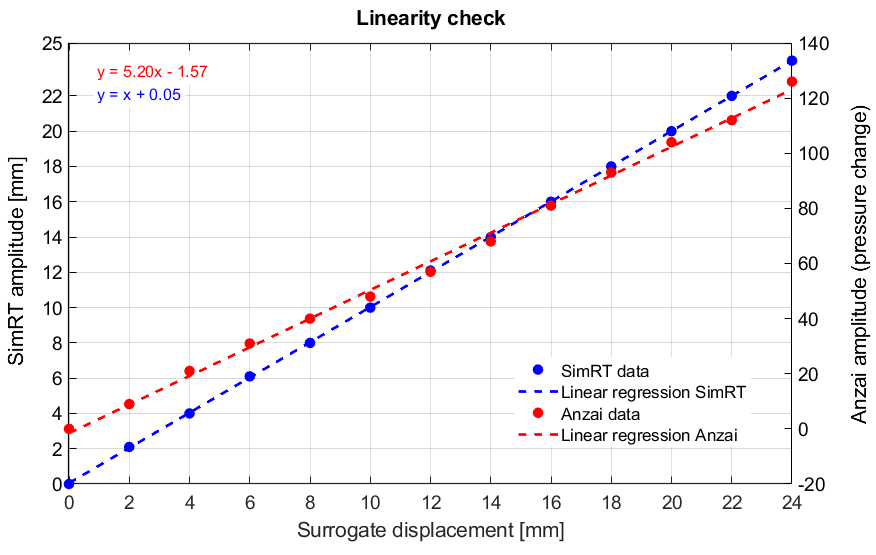


Fig. S1: SimRT and Anzai responses to applied surrogate movement in the anteroposterior (AP) direction are depicted in the graph. The linear regression fit is represented by a dashed line. The linearity was verified prior to each measurement, and the experimental setup matches that of Figure 1.


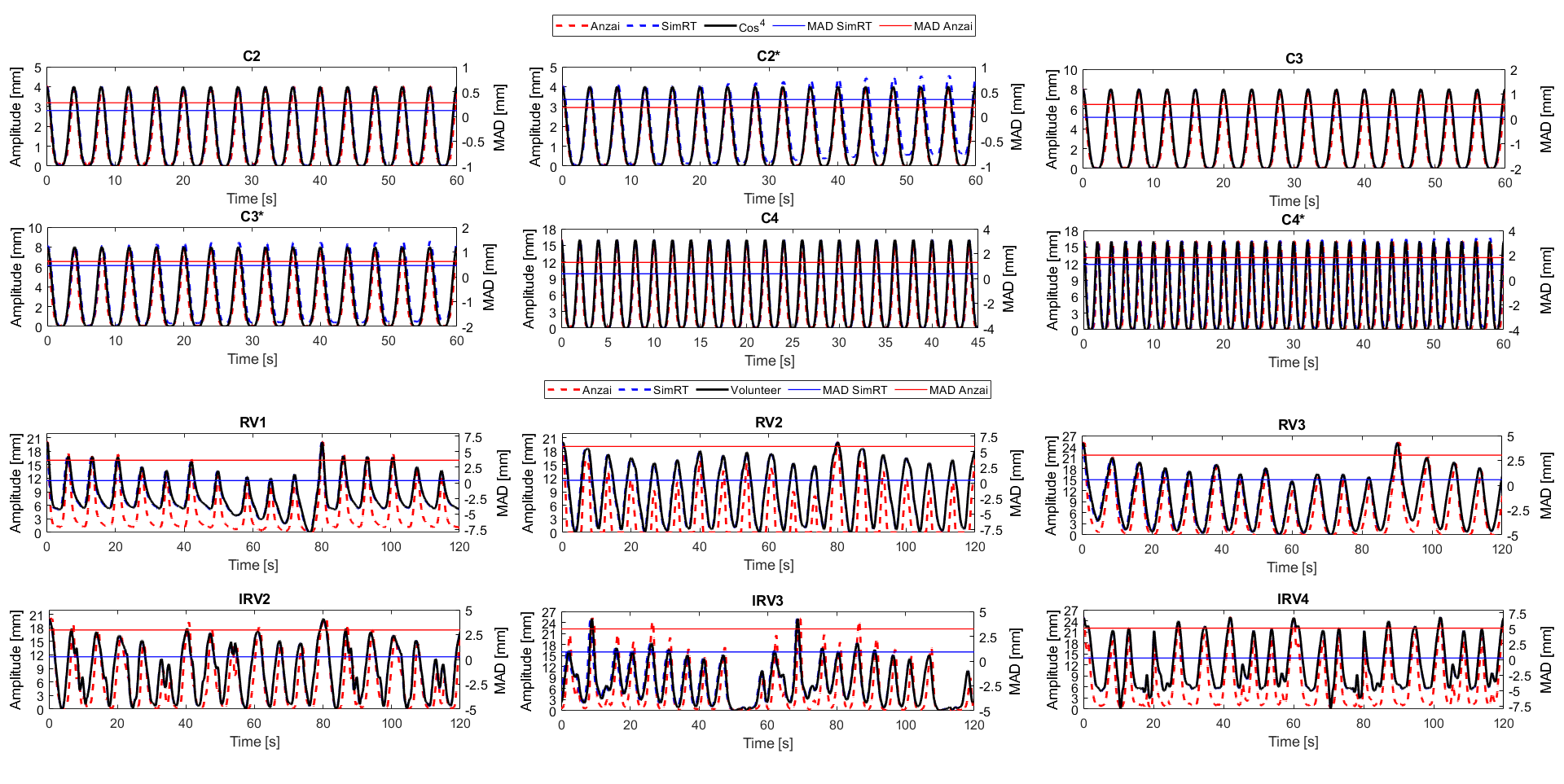


Fig. S2. Breathing patterns measured by Anzai and SimRT using the CIRS phantom compared with the ground-truth. Abbreviations: A = amplitude; CT = computed tomography; C2 = cos^4^ (A = 4 mm, without CT); C2* = cos^4^ (A = 4 mm, during CT); C3 = cos^4^ (A = 8 mm, without CT); C3* = cos^4^ (A = 8 mm, during CT); C4 = cos^4^ (A = 16 mm, without CT); C4* = cos^4^ (A = 16 mm, during CT); IRV = volunteer with irregular breathing; MAD = mean absolute deviation; RV = volunteer with regular breathing.


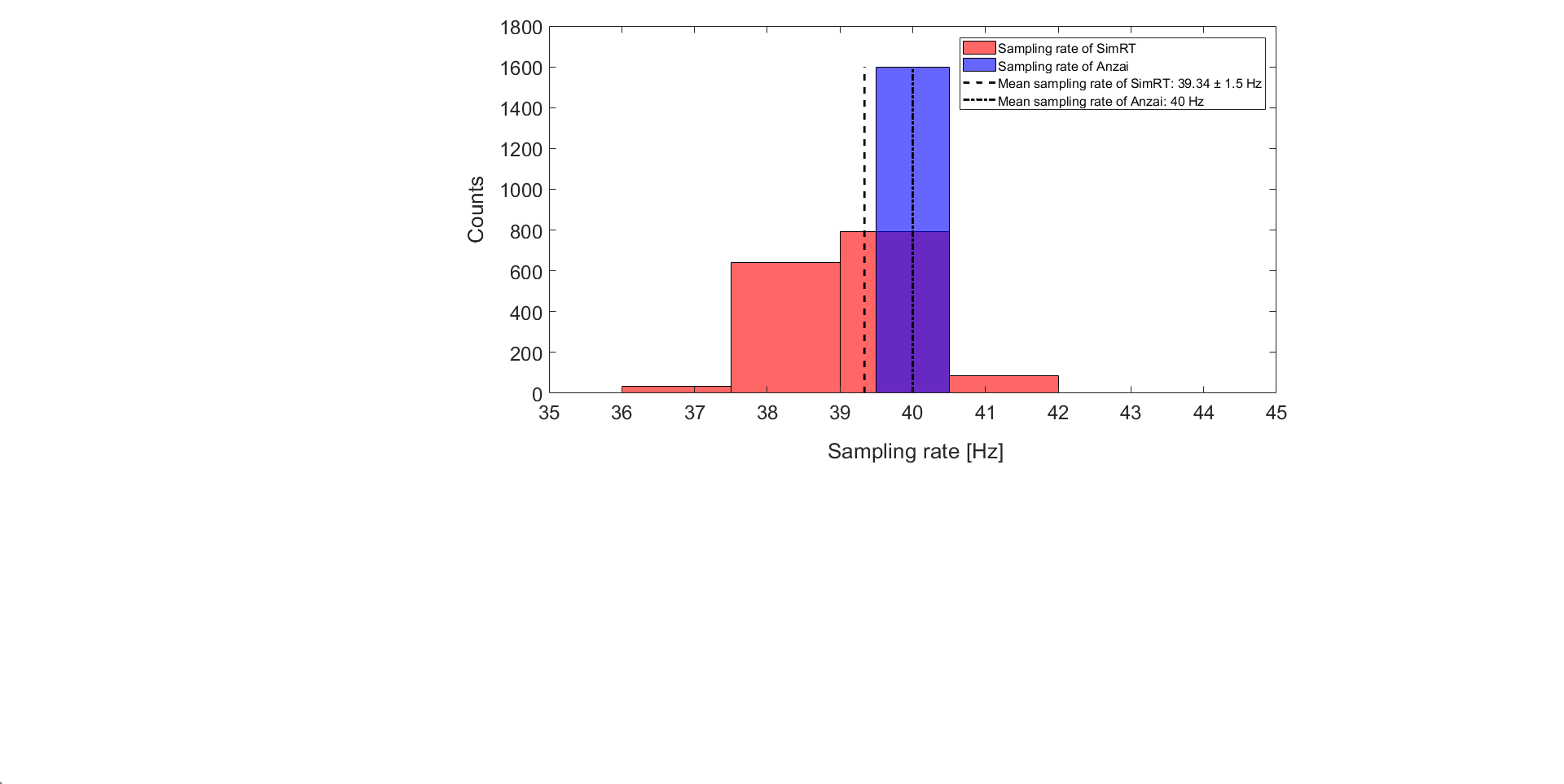


Fig. S3. Distribution of the sampling rates of Anzai and SimRT for one measurement.


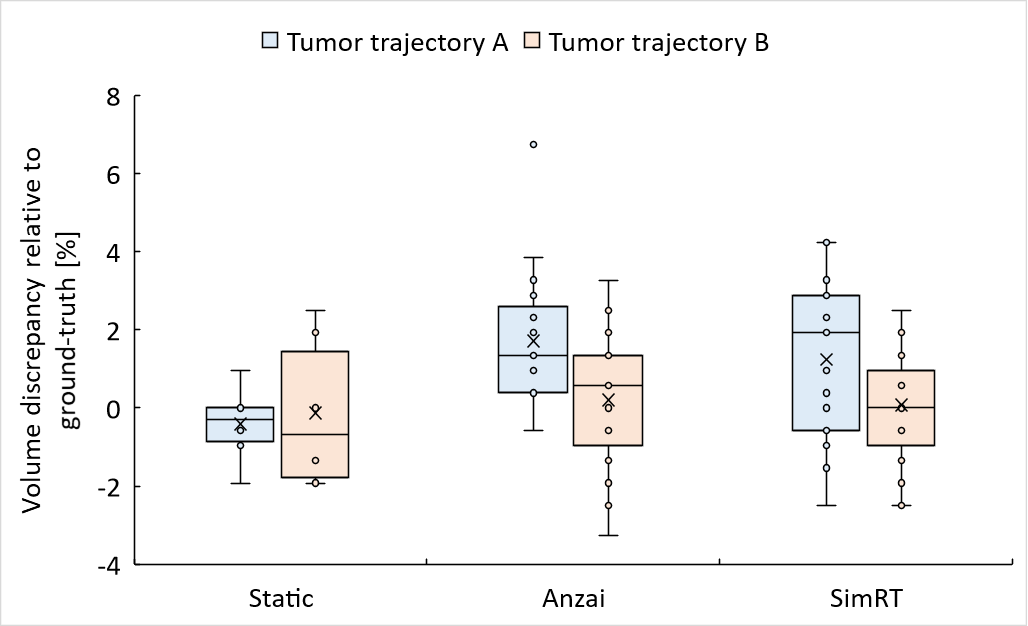


Fig. S4. Box plots with boxes extending from 25^th^ to 75^th^ percentiles showing the relative deviation in volume reconstruction for Anzai, SimRT and Static compared to the ground-truth. Relative deviation means (1-(measurement/ground-truth) * 100) %. The orange boxplots represent supplementary Table S1, and the blue represent supplementary Table S2. The x in the box plots represents the mean value. Abbreviations: Static = static 3DCT scan; 3DCT = 3-dimensional computed tomography.
